# Clinical predictors of outcome after pharyngeal electrical stimulation (PES) in non-stroke related neurogenic dysphagia after mechanical ventilation and tracheotomy: results from subgroup analysis of PHADER study

**DOI:** 10.1186/s42466-025-00380-5

**Published:** 2025-04-07

**Authors:** Ivy Cheng, Philip M. Bath, Shaheen Hamdy, Paul Muhle, Satish Mistry, Rainer Dziewas, Sonja Suntrup-Krüger

**Affiliations:** 1 Academic Unit of Human Communication, Learning, and Development, Faculty of Education, University of Hong Kong, Pok Fu Lam, Hong Kong; 2 Centre for Gastrointestinal Sciences, Faculty of Biology, Medicine and Health, University of Manchester, Manchester, UK; 3 Institute for Biomagnetism and Biosignal Analysis, University of Münster, Münster, Germany; 4 Stroke Trials Unit, Mental Health & Clinical Neuroscience, University of Nottingham, Nottingham, UK; 5 Nottingham University Hospital NHS Trust, Stroke, UK; 6 Department for Clinical Research, Phagenesis Limited, Manchester, UK; 7 Department of Neurology, University Hospital Münster, Building A1, Albert-Schweitzer- Campus 1, 48149 Münster, Germany; 8 Department of Neurology, Osnabrück Hospital, Osnabrück, Germany

**Keywords:** Dysphagia, Electrical stimulation, Mechanical ventilation, Pharynx, Tracheotomy

## Abstract

**Background:**

Pharyngeal electrical stimulation (PES) is a neurostimulation intervention that can improve swallowing and facilitate decannulation in tracheotomised stroke patients with dysphagia. The PHAryngeal electrical stimulation for treatment of neurogenic Dysphagia European Registry (PHADER) study found that PES can reduce dysphagia severity in patients with neurogenic (non-stroke) dysphagia who required mechanical ventilation and tracheotomy. However, the predictive factors for treatment success among these patients remain unclear.

**Methods:**

We conducted a subgroup analysis using data from PHADER, with a focus on non-stroke participants who had required mechanical ventilation and tracheotomy. Multiple linear regression was performed to predict treatment success, as measured in improvement in dysphagia severity rating scale (DSRS) total score, accounting for age, sex, time from diagnosis to PES, PES perceptual threshold and PES stimulation intensity at the first session.

**Results:**

Fifty-seven participants (mean[standard deviation] age: 63.6[15.5] years; male: 70.2%) were included in the analysis. These comprised traumatic brain injury (22[38.6%]), critical illness polyneuropathy (15[26.4%]), and other neurological conditions that caused dysphagia (20[35.0%]). Regression analyses identified that a lower PES perceptual threshold at the first session (*p* = 0.027) and early intervention (*p* = 0.004) were significant predictors associated with treatment success at Day 9 and 3 months post PES respectively.

**Conclusions:**

We identified two predictive factors associated with successful PES treatment in patients with neurogenic (non-stroke) dysphagia requiring mechanical ventilation and tracheotomy: a lower PES perceptual threshold at the first session and early intervention. These predictors provide critical guidance for optimizing clinical decision-making in managing non-stroke neurogenic dysphagia patients in critical care settings.

## Background

Neurogenic dysphagia is a complex problem that can negatively impact one’s physical, psychosocial, and economic well-being [1–4]. Also known as oro-pharyngeal dysphagia (OPD), it can arise from neurological injuries or conditions that impact the central or peripheral nervous systems that mediate swallowing [5]. In situations where neurological conditions lead to dysphagia-related acute respiratory failure, endotracheal intubation may be necessary as a life-saving procedure for airway protection. However, the presence of OPD can complicate these circumstances. Studies have shown that persisting intensive care unit (ICU)-acquired dysphagia after extubation (post-extubation dysphagia, PED) is a major risk factor for extubation failure [6]. Apart from the main neurological diagnosis, OPD can develop as a consequence of ICU treatment, affecting as much as 93% of patients with neurological impairments [7]. ICU-acquired dysphagia may persist after hospital discharge [8, 9] and has been found to be a predictor of 28- and 90-day mortality [10]. Tracheotomy may be performed for patients who require long-term airway support to avoid negative consequences of prolonged endotracheal intubation [11, 12]. However, the removal of the tracheotomy tube (decannulation) depends largely on the severity of OPD [13]. Therefore, OPD is a unique challenge to those with mechanical ventilation and/or tracheotomy.

Pharyngeal electrical stimulation is a neurostimulation technique that can improve swallowing in patients with neurogenic OPD through facilitating neuroplasticity changes in the human swallowing system [14–20]. In patients with oral endotracheal intubation and mechanical ventilation, PES may lower the risk of extubation failure [21], and benefit patients with PED [22]. In tracheotomised patients, PES can facilitate earlier decannulation [23, 24]. Importantly, several national and international clinical guidelines have recommended its use as a treatment for patients with post-stroke dysphagia with tracheotomy [25–27].

A recent study identified early intervention and younger age as significant predictors for PES treatment success among stroke patients who required mechanical ventilation and tracheotomy [28]. However, the factors for patients with other neurological conditions who required mechanical ventilation and tracheotomy have yet to be explored in detail. Given that the underlying pathophysiology, disease profile and progression are likely different between stroke and other neurological causes, this study explored the variables that predict success for PES treatment among patients with non-stroke related neurogenic OPD, using data collected from the PHAryngeal electrical stimulation for treatment of neurogenic Dysphagia European Registry (PHADER) study [29]. We hypothesised that specific intrinsic and extrinsic characteristics can predict better PES treatment outcomes. The findings from this study should further our understanding of factors that can influence PES treatment outcomes, which are crucial for customising intervention for maximal effectiveness among these patients.

## Methods

### The PHADER study

PHADER was a prospective observational cohort study conducted between March 2015 and September 2018 at 14 secondary and tertiary care centres in Austria, Germany and the United Kingdom [29]. A total of 252 patients were recruited initially in the study, but 7 were excluded from the analysis due to lack of consent, spontaneous recovery, unavailability of PES catheter or death. The findings from the PHADER study showed that PES reduced OPD severity and the risks of penetration and aspiration among the 245 patients with neurogenic OPD associated with stroke, other neurological conditions, mechanical ventilation, and tracheotomy. The present subgroup analysis included data collected from PHADER, with a focus on patients with non-stroke related neurogenic OPD who required mechanical ventilation and/or tracheotomy. The details of participants’ characteristics and main results have been published previously [29]. The characteristics of the participants included in this subgroup analysis, PES treatment protocol, primary outcome measure and statistical analysis relevant to this study is described below.

### Participants

**Fig. 1:**
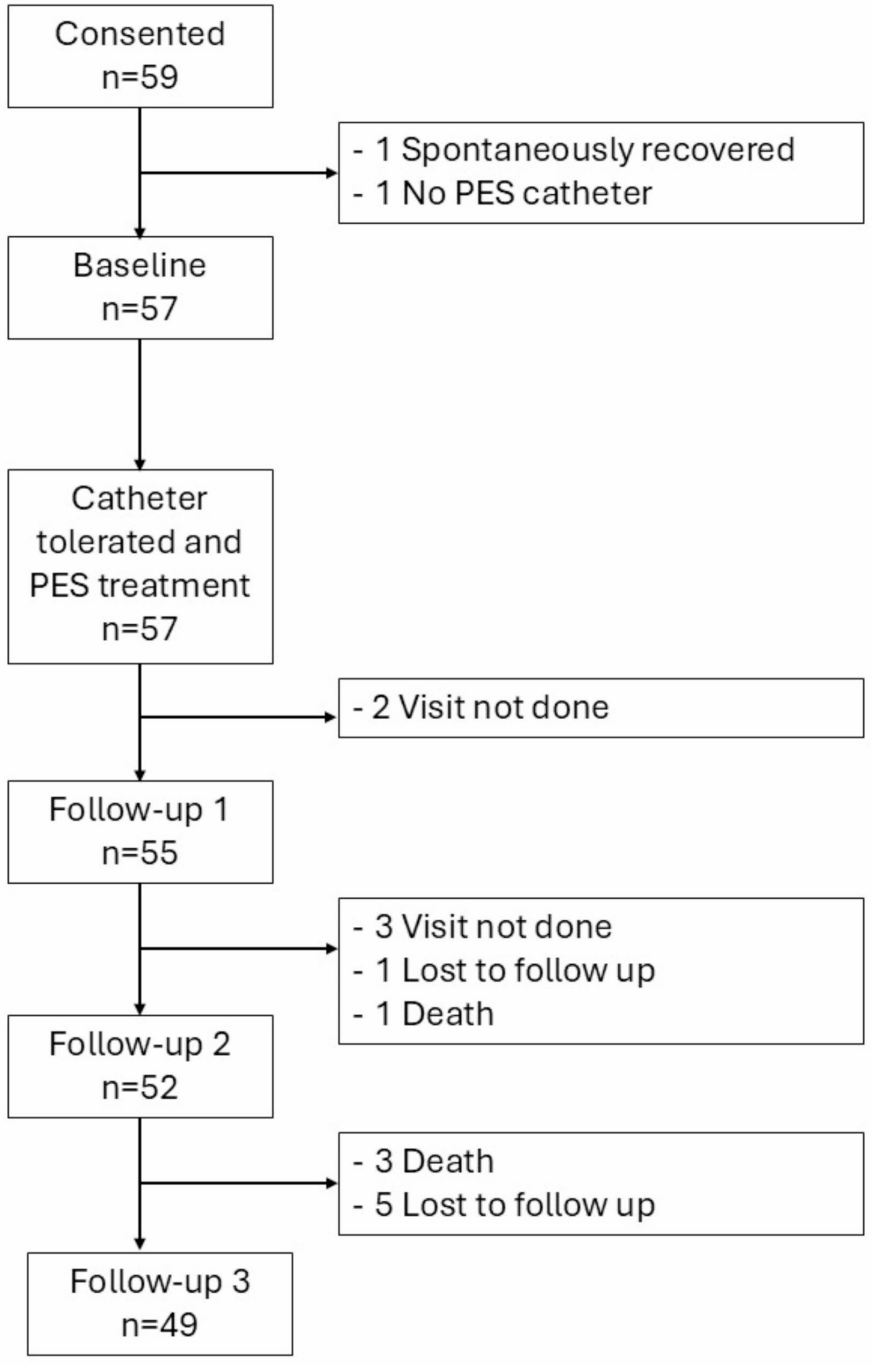
Number of participants included in this analysis. Data was extracted from the PHAryngeal electrical stimulation for treatment of neurogenic Dysphagia European Registry (PHADER) study [29]. Participants included in this analysis had neurogenic dysphagia associated with neurological conditions other than stroke

Participants who had non-stroke-related neurogenic OPD and required mechanical ventilation and tracheotomy were included in this analysis. These participants had OPD severity of 6 or above, as determined using the dysphagia severity rating scale (DSRS) [17, 30] which is a validated 13-level scale of dysphagia severity with higher values indicating more severe OPD. Fifty-nine participants consented to join the study and went through screening (Fig. 1). After screening, one participant recovered spontaneously while one did not have a PES catheter, so they were excluded from the study. Fifty-seven participants underwent baseline assessments. All of them tolerated the catheter and completed the PES treatment. The data from these 57 participants were included in this subgroup analysis.

### The PES treatment protocol

All participants received PES treatment for 10 min daily for 3 consecutive days (Phagenyx^®^ Neurostimulation System; Phagenesis Ltd., Manchester, United Kingdom). The single-patient use PES treatment catheter had built-in stimulation electrodes and was inserted through the nose to the pharynx of each participant. The catheter can also be used as a feeding tube for participants who received nasogastric feeding. PES was delivered at 5 Hz at an intensity optimised by the software and the operator. Before each treatment session, calibration was performed to determine the optimal PES stimulation intensity. During the calibration process, electric current was delivered from an intensity of 1 mA and gradually increased until the participant first felt the stimulation, referred to as “perceptual threshold”, and continued until they no longer wanted to further increase the current intensity, referred to as “maximal tolerable intensity” (50 mA at maximum). The PES stimulation intensity was then set at 75% of the difference between the perceptual threshold and maximal tolerable intensity, a parameter established in previous physiological studies [14].

### Primary outcome measure

The primary outcome measure was the total score of the DSRS [17, 30]. The OPD severity was assessed using DSRS at baseline, then again at day 5, 9 and 3 months (Day 92) after intervention. The decannulation protocol of patients with tracheotomy followed the one established in the pharyngeal electrical stimulation for early decannulation in tracheotomised stroke patients with neurogenic dysphagia (PHAST-TRAC) trial [24]. The readiness for decannulation was determined using a fiberoptic endoscopic evaluation of swallowing (FEES)-based algorithm, in which patients were assessed step-by-step for secretion management, spontaneous swallows, laryngeal sensitivity, and the risk of aspiration when swallowing jelly bolus and water [13].

### Statistical analysis

Statistical analyses were performed using IBM SPSS Statistics for Windows (Version 27.0). The predictive factors for treatment success, as measured by the change in total score of DSRS from baseline at day 5, 9 and 3 months post intervention, were identified using multiple linear regression (MLR). The variables set as predictive factors were (a) participant characteristics, including age and sex; and (b) intervention characteristics, including time from diagnosis of disease conditions that caused dysphagia to PES, PES perceptual threshold and PES stimulation intensity at the first session. All data were tested for MLR assumptions, including linear relationship between outcome variables and independent variables, multivariate normality and absence of multicollinearity, and these assumptions were not violated. A *p*-value of less than 0.05 was considered statistically significant.

## Results

### Demographics

A total of 57 participants with non-stroke-related OPD who required mechanical ventilation and tracheotomy were included in this analysis. Table 1 presents the characteristics of participants, PES treatment, and changes in DSRS total scores from baseline to each follow-up. Participants’ mean (standard deviation; SD) age was 63.6 (15.5) years. The most common neurological condition that caused OPD among these participants was traumatic brain injury (22%), followed by critical illness polyneuropathy (15%) (Table 1). All participants had severe OPD and received non-oral feeding at baseline.

**Table 1:** Characteristic of participants and pharyngeal electrical stimulation (PES) intervention and changes in the primary outcome measure at days 5, 9 and 92

	All ventilated non-stroke (*n* = 57)
**Participant characteristics**	
Age	63.6 (15.5)
Sex (Male / Female)	40 (70.2) / 17 (29.8)
*Neurological conditions causing dysphagia*	
Traumatic brain injury	22 (38.6)
Critical illness polyneuropathy	15 (26.3)
Hypoxia	3 (5.3)
Seizures	3 (5.3)
Encephalitis	2 (3.5)
Guillain-Barré	2 (3.5)
Meningitis	2 (3.5)
Tumour	2 (3.5)
Brain abscess	1 (1.8)
Cavernoma	1 (1.8)
Cerebral oedema	1 (1.8)
Encephalopathy	1 (1.8)
Multiple sclerosis	1 (1.8)
Neurosarcoidosis	1 (1.8)
Feeding status at baseline*	
Oral, normal	0 (0.0)
Oral, supervision	0 (0.0)
Oral, with support	0 (0.0)
NGT or NJT	29 (50.9)
PEG or RIG	26 (45.6)
Other routes	2 (3.5)
**Intervention characteristics**	
PES perceptual threshold at the first session (mA)	15.3 (6.0)
PES stimulation intensity at the first session (mA)	28.8 (8.9)
Time from diagnosis to treatment (days)	51.0 [48.8]
**DSRS**	
Baseline	11.6 (1.2)
Day 5	10.9 (2.5)
Day 9	9.1 (3.9)
Day 92	5.8 (5.1)

Data are presented as mean (standard deviation), number (%) or median [interquartile range].

*Feeding status at baseline was defined according to Woodhouse et al. (2018) [47]

DSRS: dysphagia severity rating scale [30]; NGT: nasogastric tube; NIHSS: National Institute Health Stroke Scale; NJT: nasojejunal tube; PEG: percutaneous endoscopic gastrostomy tube; RIG: radiographically inserted gastrostomy tube

### Predictors of treatment success among all participants who required mechanical ventilation and tracheotomy

Regression analyses revealed that lower PES perceptual threshold at the first session was a significant predictor of improvement in DSRS on Day 9 (*β* [95% CI] = 0.281 [0.034, 0.528], *p* = 0.027). The perceptual thresholds at the first session ranged from 6.0 mA to 33.0 mA, with an average (SD) of 15.3 (6.0) mA. Moreover, early intervention was another predictive factor for PES treatment success at 3 months (*β* [95% CI] = 0.013 [0.004, 0.022], *p* = 0.004) (Table 2).

**Table 2:** Multiple linear regression findings for participants with neurogenic (non-stroke) dysphagia who required mechanical ventilation and tracheotomy (*n* = 57)

Participant characteristics											
	Change in DSRS at Day 5				Change in DSRS at Day 9				Change in DSRS at 3 months		
	*β* [95% CI]	*SE*	*p*		*β* [95% CI]	*SE*	*p*		*β* [95% CI]	*SE*	*p*
Age	0.008 [-0.028, 0.045]	0.018	0.463		0.008 [-0.058, 0.074]	0.033	0.803		0.004 [-0.097, 0.105]	0.050	0.938
Sex	0.473 [-0.758, 1.704]	0.614	0.771		1.587 [-0.618, 3.792]	1.099	0.155		0.348 [-2.884, 3.580]	1.606	0.829
**Intervention characteristics**											
	**Change in DSRS at Day 5**				**Change in DSRS at Day 9**				**Change in DSRS at 3 months**		
	*β* [95% CI]	*SE*	*p*		*β* [95% CI]	*SE*	*p*		*β* [95% CI]	*SE*	*p*
PES perceptual threshold at the first session	0.091 [-0.050, 0.232]	0.070	0.202		0.281 [0.034, 0.528]	0.123	*0.027		0.199 [-0.127, 0.526]	0.162	0.225
PES stimulation intensity at the first session	-0.012 [0.106, 0.083]	0.047	0.801		-0.096 [-0.263, 0.071]	0.083	0.254		0.000 [-0.218, 0.217]	0.108	0.998
Time from diagnosis to first PES	0.001 [-0.001, 0.003]	0.001	0.343		0.003 [0.000, 0.007]	0.002	0.077		0.013 [0.004, 0.022]	0.004	*0.004

CI: confidence interval; DSRS: dysphagia severity rating scale; PES: pharyngeal electrical stimulation; SE: standard error

## Discussion

Our subgroup analysis revealed that lower PES perceptual threshold at baseline and early intervention were the two predictive factors for PES treatment success (improvements in DSRS) among patients with non-stroke neurogenic OPD. The findings provide valuable insights into intrinsic and extrinsic factors that contribute to PES treatment success in a critically ill neurogenic OPD population that is so far relatively understudied compared to stroke-related dysphagia patients.

Consistent with previous studies in stroke [23, 28], early intervention was a predictor of better PES outcomes in our mixed-aetiology OPD patient cohort. Although the pathophysiology of dysphagia and the neurophysiological mechanisms underlying its functional recovery in these diseases probably differ from each other and from stroke, impaired sensory function is a key pattern of ICU-acquired dysphagia [31] that is specifically targeted by PES, both centrally [14–19, 32] and peripherally [19, 33]. An average of 51 days from diagnosis to intervention may raise concerns for the term “early”. However, since our cohort included patients with a mixed aetiology for dysphagia, the timing of PES treatment varied among individuals, depending on their individual needs for ICU clinical treatment and when their individual clinical condition reasonably allowed PES treatment. This decision was made by the treating physician. Early intervention seems a general principle that may facilitate the recovery process and reduce ICU-acquired complications [34], e.g. pre-emptive swallowing treatments, which can be started as early as the third day of intubation, may improve swallowing efficiency in ICU patients [35]. Recent studies also suggest that early PES, i.e. delivered already prior to extubation, can significantly reduce the risk of dysphagia-related extubation failure and other complications [21, 36]. In summary, early PES treatment initiation seems generally beneficial in ICU-acquired dysphagia and should be considered in clinical decision-making.

The finding of lower PES perceptual threshold as a predictor of PES treatment success at day 9, but not at 3 month follow-up highlights the recognised importance of the sensory system as a driver for motor functional recovery of OPD [37]. When sensory input is interrupted by surface anaesthesia, the activations of sensory and motor cortical regions of the swallowing network are reduced, leading to transient OPD [38–42]. Interestingly, such induced OPD can be reversed by PES [18]. Clinical studies have demonstrated a significant association between OPD and damage to the sensory cortex and sensory deficits [43–46]. Patients with sensory deficits associated with neurological conditions may have less efficient transmission of sensory signals and ICU-treatment-related mucosa damage further impairs pharyngeal sensation. Consequently, in patients with severe oropharyngeal sensory impairments, a higher PES stimulation intensity may be required to activate sufficient sensory afferent fibres, excite the corticobulbar projections and drive neuroplastic changes for functional recovery.

Moreover, a lower perceptual threshold at baseline can be interpreted as a surrogate marker for relatively preserved pharyngeal sensory function. According to our data, these patients may show quicker improvement of OPD after PES, explaining statistical significance at day 9. Patients with worse sensory function at an initial stage may have a longer recovery process but nevertheless respond to PES treatment, so that the “perceptual threshold at first session” is no longer a significant predictor variable for treatment success at 3 months.

Our study has some limitations. Firstly, the data we analysed came from the PHADER study, which lacks a control group. However, the fast progression to improvement immediately following PES treatment in this subgroup where the treatment began at a relatively chronic stage of OPD, suggested that the PES treatment most likely explained the improvement. Moreover, the small number of patients in each aetiology subgroup precluded a more detailed subgroup analysis. Retrospective design and limited information on disease progression restricted the evaluation of confounding factors. Finally, the timing of PES treatment was dependent on the course of the underlying disease of each participant and the administration protocols of individual study sites. Therefore, there was no standardised timeline for the initiation of PES treatment across participants.

## Conclusions

Taken together, our findings suggested that in patients with neurogenic OPD, regardless of the lesion location, nature or chronicity, early PES treatment can facilitate re-establishment of neural networks necessary to drive functional recovery. Although different aetiologies may exhibit different symptoms and rates of progression, early intervention can act as a catalyst for OPD rehabilitation. Moreover, greater intactness of the sensory system is important for promoting better recovery.

## Data Availability

All data supporting the findings of this study are available within the paper, along with the published PHADER study (Bath, P. M., Woodhouse, L. J., Suntrup-Krueger, S., Likar, R., Koestenberger, M., Warusevitane, A.,… & Dziewas, R. (2020). Pharyngeal electrical stimulation for neurogenic dysphagia following stroke, traumatic brain injury or other causes: Main results from the PHADER cohort study. *EClinicalMedicine*, *28*).

## Abbreviations

CI: Confidence interval
DSRS: Dysphagia severity rating scale
ICU: Intensive care unit
MLR: Multiple linear regression
OPD: Oro-pharyngeal dysphagia (OPD)
PES: Pharyngeal electrical stimulation (PES)
PHAST-TRAC: Pharyngeal electrical stimulation for early decannulation in tracheotomised stroke patients with neurogenic dysphagia
PHADER: PHAryngeal electrical stimulation for treatment of neurogenic Dysphagia European Registry
PED: Post-extubation dysphagia
SD: Standard deviation
